# Diabetes and Risk of Cancer

**DOI:** 10.1155/2013/583786

**Published:** 2013-02-07

**Authors:** Samy L. Habib, Maciej Rojna

**Affiliations:** ^1^Department of Geriatric, Geriatric Research, Education, and Clinical Center, South Texas Veterans Healthcare System, San Antonio, TX 78229, USA; ^2^Department of Cellular and Structural Biology, University of Texas Health Science Center, 7703 Floyd Curl Drive, San Antonio, TX 78229, USA; ^3^Faculty of Medicine, Ludwik Rydygier Collegium Medicum at Bydgoszcz, Nicolaus Copernicus University, Bydgoszcz, Poland

## Abstract

Diabetes and cancer represent two complex, diverse, chronic, and potentially fatal diseases. Cancer is the second leading cause of death, while diabetes is the seventh leading cause of death with the latter still likely underreported. There is a growing body of evidence published in recent years that suggest substantial increase in cancer incidence in diabetic patients. The worldwide prevalence of diabetes was estimated to rise from 171 million in 2000 to 366 million in 2030. About 26.9% of all people over 65 have diabetes and 60% have cancer. Overall, 8–18% of cancer patients have diabetes. In the context of epidemiology, the burden of both diseases, small association between diabetes and cancer will be clinically relevant and should translate into significant consequences for future health care solutions. This paper summarizes most of the epidemiological association studies between diabetes and cancer including studies relating to the general all-site increase of malignancies in diabetes and elevated organ-specific cancer rate in diabetes as comorbidity. Additionally, we have discussed the possible pathophysiological mechanisms that likely may be involved in promoting carcinogenesis in diabetes and the potential of different antidiabetic therapies to influence cancer incidence.

## 1. Confounding Factors

Patients with diabetes are at a higher risk than the general population of developing cancer of the urinary tract, liver, biliary tract, pancreas, colon, endometrium, and kidney. Several confounding factors directly associated with clinical diversities of diabetes are varying levels of metabolic controls, duration of diabetes, profiles of antidiabetic therapy, and the presence of complications or comorbidities. Therefore it is problematic to precisely evaluate cancer risk in diabetes. Moreover, shared risk factors for both diseases such as age, sex, ethnicity, alcohol, tobacco, diet, physical activity obesity, and BMI seem to further complicate the relation [1]. Although most of studies were adjusted for this and other confounders, particular contribution of obesity, diet, and physical activity to elevated cancer rate should be taken into account. These are factors that often coexist, influence, or even cause the diabetes and have also been shown to independently influence cancer risk. The majority of diabetic patients are obese or overweight [2]. The increased cancer risk in obesity has been established in several studies for cancers of the colon, rectum, breast, endometrium, pancreas, kidney, liver, gall bladder, and adenocarcinoma of esophagus [3]. Risk ratio (RR) for cancer was increasing in parallel to a growing BMI. Obese patients with a BMI > 30 kg/m^2^ have higher RRs for cancers than overweight (BMI > 25 and <30) patients [4]. In addition to insulin resistance, obesity is known for excess estrogen production, which may elevate the risk of estrogen-dependent tumors. Notably weight gain has been shown to increase the risk of female's reproductive organs neoplasms, namely, cancers of endometrium, breast, and cervix [5–7]. 

A higher mortality rate and worsening prognosis in obese individuals diagnosed with cancer was also postulated [8], including prostate cancer that seemed to have inverse correlation with diabetes [9]. Nevertheless, as obesity is known to be accompanied by diabetes [10, 11], it is reasonable to suspect that diabetes related endocrine perturbations may be responsible for a significant proportion of obesity-cancer risk [12]. Diets with high glycemic index and high glycemic load are well established risk factors for type II diabetes [13, 14], and they have been suggested to increase risk of breast [15], pancreatic [16], colorectal, and endometrial [17] cancer. Furthermore, diets rich in fruit, vegetables, fish, and whole grain have been reported as protective from diabetes [18] as well as from neoplasms [19, 20]. These dietary modifications have been estimated to reduce risk of fatal cancer by 35% [21].

## 2. Epidemiology

### 2.1. Hyperglycemia and Cancer Risk

Several prospective studies reported an association of elevated blood glucose with increased overall cancer incidence. Impaired glucose tolerance, impaired fasting glucose, and diabetes are some factors that determine various profiles of dysglycemia. Some of them postulated linear trend of increased blood glucose levels and cancer risks, even in glucose levels still within normal, prediabetic range. Most of presented data were adjusted for sex, age, BMI, and smoking status. International study combined results from six European prospective cohorts, totaling 549,944 subjects (49% men), with mean age 45 years at baseline and a mean followup period of 11.3 years [22]. The association between glucose level and cancer was approximately linear across full range of fasting glucose levels. An increase in 1 mmol/L of fasting plasma glucose-FPG (FPG) levels was associated with RR 1.05 (95% CI; 1.01–1.10) and 1.11 (1.05–1.16) for incidental cancer in men and women, respectively. Risk ratio (RR) was 1.37 (1.14–1.64, *P* for trend = 0.002) for all-site cancer incidence in men (excluding prostate cancer) and 1.42 (1.18–1.74, *P* for trend < 0.001) in women [22].

Ten-year Korean prospective cohort study enrolling 1,298,385 subjects (64% men) showed a significant increase in all-cancer incidence for DM patients with (HRs not sure what HRs means) 1.24 (95% CI; 1.20–1.28) for men and 1.33 (1.25–1.41) for women [23]. After categorizing baseline FPG levels, linear trends in cancer incidence and mortality with increasing fasting serum glucose levels were observed in both sexes. Although almost all cases of diabetes were expected to be type II, the Korean cohort study is noteworthy for low frequency of obesity in comparison to Western population (average BMI was 23.2 and only one-fourth of participants had BMI above 25). The actual relation between hyperglycemia and subsequent cancer risk may be difficult to assess, as there are appreciable intraindividual variations in postload and fasting glucose levels [24]. Thus, random fluctuations of glucose levels over time tend to attenuate the real association between two diseases by regression dilution and in result underestimate the impact of hyperglycemia on tumor incidence [25]. Indeed, one study showed that risk of a fatal cancer was 4-fold higher after correction for regression dilution [22].

### 2.2. Glycosylated Hemoglobin (HbA_1c_) as a Marker for Cancer Risk

HbA_1c_ reflects overall glucose levels for a period of 120 days, which is the average life span of erythrocytes. As these values are not subjected to daily variations, glycated hemoglobin appears as a more objective and reliable indicator of glycaemia, particularly with regard to long prospective studies analyzing cancer incidence, where chronic, average exposure seems most relevant [26]. In 2010, HbA_1c_ levels above 6.5% were included as another criterion for the DM diagnosis [27]. A study with 46,575 participants from New Zealand was designed to evaluate an influence of glycated hemoglobin levels on cancer risk among subjects initially free of diabetes [28]. Mean age of subjects was 38 years, median HbA_1c_ level was 5.2%, and there was slight preponderance of female in study group. In the light of contemporary evidence, it remains undetermined why moderately elevated HbA_1c_ levels had stronger relation to cancer incidence than highly elevated levels. However, this study has also some relevant limitations since findings were not adjusted for obesity, and there was relative short followup period (median followup was 4.4 years) [28].

Most recent study conducted on 12,792 participants categorized in relation to HbA_1c_ levels, with median 15-year followup, published similar finding [29]. Nondiabetic women with elevated HbA_1c_ values (≥5.7%) had an increased risk of cancer incidence (HR 1.24; 95% CI; 1.07–1.44) and so did diabetic women (HbA_1c_ > 6.5%) with HR 1.30 (95% CI; 1.06–1.60). Among diabetic women, those with good glycemic control (HbA_1c_ ≤ 7%) had a 52% lower risk of cancer death than those with poor glycemic control (HbA_1c_ > 7%). Interestingly, positive association was found between HbA_1c_ < 5.0% and cancer incidence and mortality in women in Hong Kong study [30]. The increase in percentage point of HbA_1c_ was associated with 26% increase in risk of cancer (HR 1.26; 95% CI; 1.03–1.55), and use of insulin was associated with markedly lower cancer risk with multivariable HR 0.17 (95% CI; 0.09–0.32) in insulin-users group as comparing to noninsulin group. 

On the other hand, Swedish prospective cohort study evaluating cancer risk among 25,476 diabetic patients seemed to be the only large study on the field that did not confirmed previous results. HRs were all nonsignificant after comparing highest and lowest quartiles of baseline HbA_1c_ levels [31]. However, several smaller studies, investigating the risk of hyperglycemia, in relation to site-specific malignancies, reported risk of colorectal [32–34] hepatocellular [35], pancreatic [36], cancers rising parallel to HbA_1c_ values, and even to fructosamine values [37]. Distinction of studies examining risk of hyperglycemia or diabetes on cancer risk seems relevant as in studies examining the former, significant proportion of participants have already established dysglycemia (hyperglycemia) but with levels that are not eligible for diabetes diagnosis. These studies show that persons with prediabetic endocrinological dearrangements, whose prevalence in is increasing ominously, may also be affected by increased risk for cancer.

### 2.3. Diabetes and Risk Ratio to Cancer

In last decades diabetes have been consistently associated with increased risk for broad variety of malignancies. Studies reporting an increase of all cancer sites bring risk of overlooking modest association of site-specific cancers. Moreover, discrepancies on results across studies may be partially explained the various prevalence of specific cancers that represented in diverse study populations. On the other hand, increase in overall cancer incidence may not be accompanied by transparent increases in specific organ malignancies and still should be a reason for enhanced cancer surveillance among diabetic patients.

Meta-analysis that combined 12 cohort studies with a total number of 257,222 participants showed a significant elevation of pooled adjusted risk ratio (RR) for all-cancer incidence; RRs were 1.14 (95% CI; 1.06–1.23) and 1.18 (95% CI; 1.08–1.28) for men and women, respectively [38]. Another meta-analysis conducted on studies originating from Japan (with total 250,479 subjects from four cohort and one case-control study) demonstrated slightly higher total cancer risk with adjusted RR 1.25 (95% CI; 1.06–1.46) for men and 1.23 (95% CI; 0.97–1.56) for women [39]. This may be attributable to different proportion of specific cancer prevalence in Japan, with substantially higher rates of gastric, pancreatic, and hepatocellular cancers. On the other hand, subjects with diabetes receiving treatment for hypertension and/or dyslipidemia (assuming that this group reflect more intensive medical examination) showed higher HR 1.37 [40]. However, large retrospective study on total 985,815 subjects demonstrated that the risk of cancer incidence in diabetes (RR 1.56; 95% CI; 1.43–1.71) is independent of hypertension, dyslipidemia, and gout [41]. In addition another large (895,434 DM cases) retrospective cohort study [42], showed slight increase (HR = 1.19; 95% CI; 1.17–1.19) in risk of cancer for most of sites. The risk was also most influential in the younger age group making it reasonable to suspect that this may be attributable to metabolic dysfunction preceding DM diagnosis. Concurrently to previous findings, results from prospective cohort study on German population (26, total of 742 subjects) showed an increased risk of cancer with SIR 1.14 (95% CI; 1.10–1.21), but the duration of diabetes was inversely associated with the cancer risk which was markedly higher within the first year after diabetes diagnosis [43].

Observations from two retrospective cohort studies from Belgium (4012 diabetic subjects) and China (7,950 diabetic subjects) were consistent with preceding reports, although the overall risks for cancer were slightly higher with HR 1.84 (95% CI; 1.51–2.24) [44] and SIR 1.331 (95% CI; 1.143–1.518) in men and 1.737 (95% CI; 1.478–1.997) in women [45]. According to inclusion criteria of most of studies, risk of cancer was evaluated among DMII patients, and cases of nonmelanoma skin cancer were excluded from the analysis. In summary, diabetes and hyperglycemia were shown to be associated with elevated general cancer risk. Exact values of estimated risk may vary because of study design, impact of confounding factors, and ethnic differences including genetic susceptibility, life-style behaviors, specific environmental exposures, and varying biological effect of diabetes across populations. All these factors are summarized in several cohort, prospective, and meta-analysis studies from different countries (see Table 1 for more details).

## 3. Site Specific Cancer Risk in Diabetes

### 3.1. Liver

Hepatocellular carcinoma (HCC) is the second most frequent cause of cancer death in men worldwide. In addition to pancreatic cancer it has been studied most extensively in regard to DM as well as the association between DM, and those malignancies have been reported as the strongest [46]. Recently conducted meta-analysis of 25 cohort studies indicated that diabetes mellitus was associated with significantly increased risk of HCC (combined SRRs = 2.01; 95% CI; 1.61–2.51) as comparing to subjects without diabetes [47]. Risk was independent of geographic location, alcohol consumption, history of cirrhosis, or infections with HBV or HCV. Although exact mechanisms underlying the link of diabetes and HCC are not clear, some hypotheses have been put forth. Hyperinsulinemia and resulting increase of IGF-1 can stimulate cellular proliferation, inhibit apoptosis, and promote carcinogenesis. This view has been supported by *in vitro*, animal model, and epidemiologic studies [48, 49]. Nevertheless due to insulin secretion burst to portal circulatory system, healthy liver cells are physiologically exposed to 3–9-fold higher insulin concentration, comparing with peripheral tissue [50]. Thus direct mitogen action of insulin seems less probable than in other organs. 

Diabetes may also act synergistically to other well-established risk factors for HCC, since HCV-positive persons, were more than three times more likely to have TIIDM [51], and HCV core protein has been shown to induce insulin resistance [52, 53]. Furthermore, *in vivo* and *in vitro* studies revealed that chronic and acute alcohol consumption can produce insulin resistance in liver [54, 55]. Though, most plausible link seems to arise from nonalcoholic fatty liver disease (NAFLD), the most common chronic liver disease in the Western world which is also considered as hepatic manifestation of diabetes and metabolic syndrome, as several studies consistently report nearly 70% prevalence of NAFLD in diabetic patients basing on ultrasonography and liver biopsy [56]. Liver cancer can progress in some patients from simple steatosis to inflammation and develop nonalcoholic steatohepatits (NASH), which prevalence among diabetics was estimated to be 22.2% [56]. NASH can lead to fibrosis, cirrhosis, and hepatocellular carcinoma, while this sequence usually has not been accompanied by any symptoms, signs, or even elevation of liver enzymes [57]. NAFLD has been shown to be a potential contributor to a significant proportion of cryptogenic cirrhosis cases [58–60], which are responsible for 30–40% of HCCs in Western societies [61]. HCC may arise from NASH without preexisting cirrhosis stage or even from NAFLD with mild or absent fibrosis [61, 62]. In a German study, analyzing 162 cases of HCC, NAFLD was found as an underlying etiology in 24% patients [63]. Reassuringly, these tumors tend to be well differentiated, solitary, and larger with better prognosis [61]. 

### 3.2. Pancreas

Recent meta-analysis comprises 35 cohort studies addressed diabetes as a risk factor for pancreatic cancer with combined RRs = 1.94 (95% CI; 1.66–2.27), independently of geographic locations, sex, study design, alcohol consumption, BMI, and smoking status [64]. However, given the negative correlation of duration of DM with pancreatic cancer risk, the controversy regarding the causal role of diabetes has risen. The highest risk of pancreatic cancer among studies was found within the first year followup (RRs 5.38), and then it gradually decreased as the duration of diabetes of 1–4 years conferred higher risk than DM lasting from 5 to 9 years, with RRs 1.95 and 1.49, respectively [64]. Hence, the theory of reverse causation has been established where pancreatic cancer can induce a diabetic state and is supported by following findings. The frequency of diabetes diagnosis among patients with pancreatic cancer has gradually and continuously increased for 3 years preceding cancer detection [65]. Similar tendency were found regarding FPG levels, with an inverse relation to BMI [66]. Diabetes, predominantly new onset, was estimated to have more than 40% prevalence among pancreatic cancer [67]. Moreover, the majority of new onset diabetes cases resolved after surgical resection of pancreatic tumor [67–69]. The occurrence of hyperglycemia was independent of pancreatic cancer stage and site [67], and a 2030 MW peptide was identified as a potential diabetic factor released by pancreatic cancer cells [70]. Thus pancreatogenic diabetes (Type 3c DM) should be discriminated from new onset type II DM, based on a negative family history of DM, recent weight loss >2 kg, premorbid or usual BMI < 25 kg/m, and age 65 years and older [71]. Absence of elevated pancreatic polypeptide (PP) serum levels after nutrient ingestion was proposed as reliable clinical test for T3cDM [72], which may become a sufficient indicator for implementing high-resolution imaging of the pancreas in these patients, as it was considered not practical to pose such an indication relying on new onset diabetes by itself. However, the possibility that longstanding diabetes is an actual risk factor cannot be excluded. After 10 years of diabetes, moderately elevated risk of pancreatic cancer remained with RRs 1.47 [64]. In TIIDM, exocrine pancreatic cells are exposed to unparalleled high insulin levels given their proximity to insulin secreting islets, so hyperinsulinemia may likely account for the risk in the setting of direct growth promoting features of insulin and highly expressed IGF-1 and insulin receptors in pancreatic cancer cells [73, 74]. Furthermore, markers of pancreatic ductal replication were increased 4-fold in lean type II diabetic subjects and 10-fold in obese nondiabetic subjects. Expression of the same markers was markedly higher in samples of pancreatitis, pancreatic tissue surrounding pancreatic cancer and from obese diabetic subjects [75]. Epidemiologic studies indicated that HbA_1c_ [36], FPG, and insulin levels were proportionally associated with pancreatic cancer risk, which was the highest after a 10 years of followup [76]. 

### 3.3. Colon

Most recent meta-analysis comprising 24 studies (8 case control and 16 cohorts) linked diabetes with modest increase in risk of colorectal cancer (CRC), the most common cancer of digestive tract in western societies, with RRs 1.26 (95% CI; 1.20–1.31) [77]. The risk did not differ significantly by sex and subsite, but previous meta-analysis which presented similar results pointed that longer duration of DM (11–15 years) had strongest influence on CRC incidence [77]. Several studies showed that increased levels of IGF-1, insulin and elevated HbA_1c_ (>7.5%) were all associated with higher occurrence of adenomatous polyps that presented at younger age [78–80]. It was suggested also that slower bowel transit and constipation, which are more common among diabetics, may lead to prolonged exposure of colon mucosa to toxins and potential carcinogens [81]. For example, higher concentrations of fecal bile acids were associated with intramucosal adenocarcinomas [82, 83].

### 3.4. Other Organs

Meta-analysis of 15 trials (10 case control and 5 cohort) evaluating risk of cholangiocarcinoma (CC) in diabetes (included intra- and extrahepatic locations) indicated summary RRs 1.60 (95% CI; 1.38–1.87) in DM [84]. Diabetes-related CC risk may be mediated by increased formation of biliary stones, a known risk factor for CC, while diabetes and insulin resistance was shown to be independently associated with gallstones formation [85]. In addition, diabetes was associated with modest increase of esophageal carcinoma (SSRs 1.30; 95% CI; 1.12–1.50), according to meta-analysis of 17 studies (6 case control and 11 cohorts) [86]. Esophageal carcinoma consists of two main histological subtypes: squamous cell carcinoma (ESCC) and adenocarcinoma (EAC). Interestingly, reflecting three case-control studies, which distinguished EAC as an outcome, combined SRR for adenocarcinoma was 2.12. It seems more alarming in the context of continuing rise in incidence rates of EAC, that now is estimated to account for 30–50% of all EC cases comparing to only 5% two decades ago [86]. Above findings may be attributed to delayed gastric emptying and subsequent dyspepsia, that are more frequent in diabetes and that simultaneously can lead to Barrett's esophagus and adenocarcinoma [87].

Nine cohort studies were included in meta-analysis assessing the kidney cancer risk in diabetes showed that RR of kidney cancer in diabetes was 1.42 (95% CI; 1.06–1.91). The association was stronger in women (RR = 1.7; 95% CI; 1.47–1.97) [88]. Frequent comorbidities of diabetes such as hypertension and end stage renal disease (ESRD) may mediate its risk for kidney cancer, as those states were also documented as risk factors for kidney malignancy [89–91]. Moreover, recent association between chronic kidney disease (CKD), the lesser stage of ESRD, and cancer was established [89]. Diabetes remain the most common cause of CKD and ESRD [92]. Also liver, lung, and in particular urothelial cancer are strongly associated with CKD, with the risk of neoplasms increasing parallel to renal function decline measured by GFR. Impaired excretory renal function in CKD results in higher circulating levels of carcinogens and toxins and immune inhibition, factors that may underlie the CKD-cancer relation [89]. Examination of tumor nephrectomy specimens revealed a wide array of histopathological changes in noninvaded kidney parenchyma, as only 10% of cases had normal renal tissue adjacent to tumor [93]. Diabetes related pathologic alterations (glomerular hypertrophy, mesangial expansion, and arteriolar hyalinosis) were second most frequent changes detected in nonneoplastic renal tissue of tumor nephrectomy samples [93, 94]. Recent study from our lab shows that 25.4% of kidney cancer patients have diabetes considering a number of variables including ethnicity, age, and severity of the disease at the time of diagnosis [95].

Findings from meta-analysis of 16 studies suggest that individuals with diabetes may have a modestly increased risk of urothelial cancer (RR = 1.24; 95% CI; 1.08–1.42) [96]. In line with this results, subsequent large prospective multiethnic cohort study [97] (186,000 participants from five ethnic group) reported similar RR = 1.25 (95% CI; 1.04–1.50) over a median 10.7 years of followup. While kidney cancers arising in the native kidneys of CKD and ESRD patients tend to have rather benign clinical course [98], increasing severity of CKD resulted in more aggressive and advanced urothelial cancers [99, 100]. Similarly, diabetes was recognized as independent predictor of worse outcome after urothelial cancer diagnosis [101]. Risk of urinary tract infection is elevated in diabetes, and the former was also linked to bladder cancer risk [102].

Breast and endometrial cancer patients were also more likely to have a history of diabetes. Meta-analysis reflecting breast cancer risk (5 case-control and 7 cohort studies) demonstrated moderate association between breast cancer and diabetes with summary RR 1.25 (95% CI; 1.20–1.29) [103], whereas another meta-analysis (3 cohort and 13 case-control studies) reported more than two-fold increase in risk (RR = 2.1; 95% CI; 1.75–2.53) of endometrial cancer in diabetic women [104]. However, such high-risk estimate may be due to the preponderance of case-control studies in latter meta-analysis. Interestingly, according to subset analysis of three studies, type I diabetes was found to strongly affect the cancer risk, with summary RR 3.15. In addition, hyperinsulinemia is associated with excessive ovarian androgen secretion and decreased levels of circulating sex hormone binding protein (SHBG), which leads to higher concentrations of bioactive estrogens, that are known as risk factors for malignancies of female reproductive organs [105, 106]. Hematologic malignancies also seem to be somehow associated with diabetes. Meta-analysis of 26 trials reported higher risk of non-Hodgkin lymphoma (particularly peripheral T-cell lymphoma), leukemia, and myeloma, but not Hodgkin lymphoma among diabetics [107].

In general, risk for malignancies in diabetes is varying regarding specific organs, which is expected in the sight of complexity of metabolic perturbations underlying diabetes, its influence on different organs, and distinct biology of each tumor. It appears that diabetic patients are at particularly greater risk of liver, endometrial, and pancreatic cancer. As the vast majority of studies accounted for type II diabetes (by excluding sudden onset diabetes as well as diabetics under 30 years of age) risk of cancer in type I diabetes remains unclear. According to recent systematic review, evidence on increased cancer incidence specifically in type I DM is limited and inconclusive [108]. However, risk of certain tumors, like pancreatic and endometrial cancers, is likely increased in type I DM, as it was pointed in meta-analyses [104, 109].

Data in Table 2 summarized the link between all type of cancers and diabetes in recent published cohort and case studies.

## 4. Misclassification of Exposure

In vast majority of studies after exclusion of first 2 years of followup, the risk remains significantly increased. Reversal causality, meaning that the malignant process is leading to diabetes, appears to exist in pancreatic cancer, but again it does not account for the entire risk, so both effect and cause relation can be distinguished. In addition, hyperinsulinemia reflects some degree of causality in carcinogenesis, while risk of cancer may be expected rather to diminish with long-term type II DM since levels of endogenous insulin fall down due to beta cells failure. On the other hand, the actual magnitude of diabetes-cancer relation may be underestimated, as considering the following issues. Diabetes is an underdiagnosed disease, as nearly one-third of those having diabetes are undiagnosed [110]. In studies relying on self-reported DM, some subjects in controls group may also have diabetes. Prevalence of DM among controls group increased over time, and in some studies baseline inclusion criteria for diabetes had higher cut off values than current ones. Furthermore, it is of note that insulin plasma levels are highest in the period directly preceding diabetes, development of diabetes is typically an insidious process, extending over many years, and a significant proportion of patients are either undiagnosed of diabetes or have prediabetes (35% prevalence among persons over 20 years) [110].

Hence, while process of insulin resistance and other metabolic abnormalities accompanying diabetes are already established, the potential malignant influence of diabetes may affect greater proportion of population. Indeed, according to some studies, the highest cancer risks were found among subjects just below the threshold commonly accepted for DM diagnosis [28, 111]. Additionally some studies tend not to distinguish between type I and type II DM relying on fact that estimated 95% of cases are type II diabetes [110]. This may also slightly attenuate the risk of cancer in DMII, as DMI was shown to have no or very weak association with cancer [108]. In order to reduce the risk of cardiovascular disease, diabetic patients are more likely to receive aspirin that lowers risk of cancer [112–114]. Studies performed previously to “aspirin era” reported higher risk of cancer in DM. 

Diabetes was shown to act synergistically with other definite cancer risk factors as HP infection for gastric cancer [115]. Also risk of pancreatic cancer in chronic pancreatitis was dramatically increased with coexistence of diabetes (HR = 33.52), even though the role of DM may be overlooked in the presence of more acknowledged risk factors [116]. Despite potentially more frequent interaction with health care system, several studies consistently reported lower rates of screening for breast [117], cervical [118], and colon cancers [119]. Finally, risk of cancer of several organs may be also ascribed to frequent DM comorbidities, while their occurrence is in fact strongly determined by diabetes. This is attributable to kidney cancer, ESRD, CKD, and liver cancer that may arise from the spectrum of NAFLD, NASH, and subsequent cirrhosis. Diabetes is leading cause of both, CKD, and NAFLD.

## 5. Diabetes Drugs

Considering the diabetes and cancer association, the most direct concern for patients as well as clinicians is that different treatment arms seem to modify the risk of cancer. Due to progressive clinical course, management of DMII is changing over time and is being adjusted to each patient individually. 

### 5.1. Metformin

Metformin, an insulin sensitizer, is a first line and most commonly used drug in management of diabetes, either as initial or combination therapy. Recently, metformin has attracted special attention as strong body of evidence from epidemiologic, *in vitro,* and *in vivo* model studies reported its antineoplastic properties [120–122]. Most recent meta-analysis of seventeen observational studies demonstrated that metformin was associated with 39% lower cancer risk (summary RR 0.61; 95% CI; 0.54–0.70) as comparing to reference therapies (insulin, sulfonylureas, or no medication) taken together [123]. Several classes of mechanisms are proposed to contribute beneficial role of metformin with regard to malignancies. Metformin inhibits complex I of mitochondrial electron transport chain and thereby attenuates oxidative respiration resulting in ATP/AMP imbalance, which in turn activates LKB1 and AMP-kinase (AMPK). Downstream signaling pathway of AMPK comprising mTOR inhibition, cyclin D1, and p53 interference most likely accounts for antiproliferative effects of metformin. Furthermore, metformin was shown to repress proliferation and survival of cancer stem cells as well as restrain the process of epithelial-mesenchymal transition (EMT), where cancer cells are acquiring metastatic phenotype [121, 124]. Additionally, reducing systemic risk factors by indirect mechanisms like lowering glucose, insulin, and insulin receptors levels along with immunosuppressive properties has been pointed out [122]. Similarly, metformin may lead to caloric restriction conditions [125] known for preventing from tumor occurrence [126]. Finally, metformin is capable of reducing endogenous ROS generation therefore impeding mutagenesis in somatic cells [127]. Besides lowering incidence rate, metformin was reported to diminish cancer mortality [128]. In addition, metformin improved chemotherapy outcome in breast cancer patients [129], lung cancer patients [130] and was recognized as independent factor of colorectal cancer survival [131]. Moreover, metformin amplified anticancer effect of chemotherapeutic agents as doxorubicin [124] and paclitaxel [132].

### 5.2. Thiazolidinediones

Other classes of compounds used in DM therapy have less transparent link to neoplastic disease. *In vitro* studies reported potentially anticancer properties of thiazolidinediones, insulin sensitizing PPAR-gamma agonists [123, 133, 134]. Nevertheless, *in vivo* and epidemiologic trials presented conflicting evidence [135]. Meta-analysis of randomized control trials (RCT) showed that rosiglitazone did not modify overall cancer risk [136] (OR 0.91 (95% CI 0.71–1.16), *P* = 0.44); however, meta-analysis of studies investigating specifically bladder cancer reported elevated risk of this neoplasm in thiazolidinediones users according to cohort studies, which was not confirmed by two RCTs included in the study [137]. Most recently thiazolidinediones therapies known for positive influence on blood pressure, lipid profile, and weight reduction were linked to thyroid and pancreatic malignancies [138].

### 5.3. Insulin Secretagogues

In terms of ongoing debate on role of hyperinsulinemia in tumor genesis there is a growing concern that drugs translating into increased levels of circulating insulin may represent a risk factor. Indeed several trials documented that use of insulin secretagogues particularly sulfonylureas, is associated with higher cancer incidence and mortality [139–141]. On the other hand, meta-analysis did not confirmed this data showing neutral effect of sulfonylureas on cancer incidence [142].

### 5.4. Insulin

Eventual initiation of insulin treatment after failure of oral drugs to maintain proper glycemic control was necessary in 40–80% of diabetic patients [143]. Of note, while physiologic pattern of insulin secretion to portal vein included degradation and utilization of significant proportion of the hormone in the liver, exogenous administration confer higher availability of insulin to peripheral tissues. Insulin therapy was associated with higher risk of malignancies as stated in recent meta-analysis (summary RR = 1.39; 95% CI; 1.14, 1.70) [144]. Regarding the insulin glargine controversy, several methodological flaws and limitations were pointed out in the study that raised the concern [145–147], and meta-analysis of trials comparing glargine to other insulin showed neutral effect of glargine on cancer incidence [148]. However, it may be problematic to determine the actual influence of certain therapies on cancer risk as they are compared to agents potentially lowering the risk, hence protective effect of one drug may be confounded to be pro-carcinogenic features of other drugs. Moreover, clinical profile of metformin-treated diabetics is distinct from those using other therapies; namely, biguanides are prescribed more often to newly diagnosed patients free of other serious comorbidities like kidney, lung, heart, or liver diseases, whereas insulin tends to be introduced to older patients with longer duration of DM. Nevertheless, as to metformin, evidence regarding its tumor protective effects remains strong and convincing. It is also relevant to stress that cardiovascular risk remains a major threat for diabetic patients, thereby optimal glucose control, which reduces the risk of diabetes-associated complications, should be a central goal of proper diabetes management.

## 6. Mortality

Studies relating to survival evaluate cancer outcome comparing cancer patients with DM and cancer patients without DM, whereas studies referring to mortality examine cancer related deaths among diabetic or nondiabetic group. In addition to increased incidence of cancer, DM diagnosis has also deleterious effects on cancer prognosis. It is relevant to distinguish studies assessing mortality and survival rates in cancer patients with DM. Higher mortality rates, to some extent, may be secondary to increased incidence of cancer in DM. Meta-analyses of 12 studies reported higher mortality across all cancers types in diabetes (RR 1.16; 95% CI; 1.03–1.30) [38]. Additionally decreased short-term survival rates were noted among diabetics undergoing surgical resection of colorectal cancer [149] hepatectomy due to colon cancer metastases [150] as well as esophagogastrectomy [151, 152]. Most importantly, diabetics with cancer had lower rates of long-term all-cause survival than cancer patients without DM according to the meta-analysis of 23 studies (HR; 1.41; 95% CI; 1.28–1.55) [153].

 It is reasonable to address the question by which mechanism and pathways diabetes influence the poorer cancer prognosis. Extensive explanations are provided elsewhere [154]. Diabetes may result in more aggressive clinical course of cancer, strengthening its metastatic potential, favoring cancer growth by making host organism less resistant to cancer progression, possibly by known impaired immune function in diabetes. Hyperglycemia may more likely be involved in tumor progression by favoring growth of cells owing to Warburg effect, than in initiation of malignant process. Epidemiologic studies documented that survival rates in cancer are decreasing linearly to declining glycemic controls [155, 156]. DM patients with cancers of ovary, colon [157], prostate [158] but mainly breast [159, 160] were reported to present at more advanced stage. This is probably largely due to lower screening usage of diabetic patients, especially mammography [117]. In the setting of diabetes and related metabolic perturbations, chemotherapy agents may be less efficient [129], and recurrence rate might be higher after radiation or surgery [161, 162]. Also, less intensive and aggressive treatment regimen is likely offered to DM patients in the context of already elevated risk of cardiovascular events and development of kidney, liver, and heart failure. 

DM patients, more often are poor candidates for surgery [163] and oncologist may administer lower doses of anticancer agents to avoid occurrence of adverse effects [157, 160]. The latter is probably also attributable to higher chemotherapy related toxicity in DM patients. For example, in colon cancer patients with DM, there was a greater risk of postoperative hepatic decompensation after surgical removal of liver metastases [150] or higher occurrence of diarrhea after 5-fluorouracil adjuvant chemotherapy [162]. In breast cancer patients diabetes predicted a whole range of chemotherapy toxicities increasing risk of all-cause hospitalization [160]. Moreover, some of anticancer treatment regimens, including glucocorticosteroids and androgen deprivation therapy [164] or mTOR inhibitors [165, 166] used in advanced renal cell carcinoma tend to aggravate glycemic control, thereafter increasing risk of cardiovascular disease. Finally, diabetes is associated with higher all-cause mortality [167]. Common specific causes of death in cancer are cachexia, thrombosis, pneumonia, and opportunistic infections. DM can independently impose risks of those particular diseases, so higher cancer mortality rates in DM may be more attributable to DM by itself than to more aggressive tumor behavior under diabetic conditions. 

## 7. Discussion

Diabetes significantly increases risk for several cancers, as well as it negatively affects the prognosis after cancer diagnosis. Relative risk imposed by DM is greatest (about 2-fold or higher) with regard to liver, pancreas, and endometrium cancer and lower for cancers of kidney, bladder, breast, colorectal, esophagus, biliary tract, and lymphoma. It appears that there is a very small risk for gastric and lung cancers, while the risk for prostate cancer is decreased with a history of DM. Diabetic patients with cancer have lower short- and long-term survival rates than their nondiabetic counterparts. It is highly possible that cancer's adverse effects on thrombosis and oxygenation, immune response as well as the cardiovascular risks imposed by cancer surgery, may be amplified by diabetes, which independently contribute to increased risk of those states.

Since cancer and diabetes are heterogeneous, multifactorial diseases with complex background, several potential pathophysiological pathways can contribute their interdependence. Apart from hyperglycemia and insulin resistance, diabetes is further characterized by a low-grade chronic inflammation, reduced antioxidant capacity, dyslipidemia, hypertension, procoagulation, adipose tissue expansion with adverse secretory profile of cytokines, and altered hormonal concentrations [168]. To add dimensions to this complexity, the relation between diabetes and cancer may be modulated by various genetic factors among ethnic groups, differences in lifestyles, and environmental exposures.

Hyperinsulinemia is a compensatory response for increasing insulin resistance of liver, muscles, and adipose tissue. Overt diabetes occurs when pancreatic *β* cells are no longer capable of intensive insulin production. Numerous epidemiological studies reporting correlation of elevated C-peptide, postprandial, and fasting insulin levels with cancer risk were summarized [169] and meta-analyzed [170]. Significantly increased risk of colorectal, pancreatic, breast, and endometrial cancers was found in highest categories of insulin/peptide C levels [170]. Insulin is a potent growth factor and can exert proliferative and carcinogenic influence in various manners, directly or through IGF. Hyperinsulinemia leads to increase of bioavailable IGF by inhibiting IGFBP1 in liver and may also sensitize cells to this hormone. *In vitro* studies confirmed growth promoting and apoptosis inhibition properties of insulin and IGF in physiological concentrations [171, 172]. Hyperinsulinemia may also facilitate tumor cell migration [173]. Moreover, insulin and IGF receptors are broadly expressed by normal tissue as well as cancer cells [12]. Expression of IR-A receptor which has more pronounced mitogenic than metabolic effect is expressed more commonly [174]. Besides, insulin resistance confers failure mainly of metabolic cell response, while downstream mutagenic signaling effects are preserved [175]. Although early phase clinical trials demonstrated that (IGF1R) specific antibodies are increasing sensitivity of colon cancer stem cells to chemotherapy, phase III results were unsatisfactory [175].

Hyperglycemia may serve as another plausible explanation. Strong epidemiological evidence indicates that high sugar intake and elevated serum glucose levels are associated with higher cancer incidence and mortality [176]. Animal model hyperglycemia was shown to be involved in metastatic processes [177], and liver tumors tend to be significantly larger in high glucose environment which was independent of hyperinsulinemia [178]. Enhanced formation of advanced glycation end products (AGE) was documented as a growth promoter for melanoma, pancreatic and possibly colon cancer in animal models [179]. Hyperglycemia may exert a direct tumor promoting effect, since cancer cells rely strongly on glycolysis [180]. Although generation of ATP requires by far more glucose than oxidative phosphorylation (feature which is used in PET imagining), malignant cells are already adapted to highly effective glucose uptake independently of insulin signaling, so that additional favorable effect of hyperglycemia is unclear. 

Apart from direct actions of insulin and high glucose, various metabolic dearrangements, parallel to DM, can contribute to development of tumor. TIIDM is perceived as achronic proinflammatory condition [181–183], and inflammation is considered as a hallmark of carcinogenesis [184–186]. TNF alpha, IL6, and PAI1 may act as bridging molecules, while they are intensively secreted by adipose tissue [185], and involvement of each was described in malignant transformation [187–189]. Moreover, hyperglycemia by increased formation of AGE can stimulate NF*κ*B production and in turn cancer promotion [190]. Imbalance between production of reactive oxygen species (ROS) and cellular utilization capability result in oxidative stress which can be induced by diabetes [191] and chronic inflammation [192]. Oxidative stress leads to genomic instability, DNA mutations, and it may be attributed to wide range of cancers [192].

Hyperglycemia by itself can induce DNA damage [193], downregulation expression of antioxidants [194], and increase ROS generation [195, 196]. Since glucose and fat metabolism are closely related, some authors investigated the potential correlation between certain range of dyslipidemic parameters and cancer risk in diabetic patients. Low levels of LDL and triglyceride, high HDL, and copresence of low LDL with albuminuria were significantly associated with cancer risk in TIIDM [197, 198]. Epigenetics refers to the regulation of gene expressions without changes in nucleotide sequence, therefore allowing for phenotype modification without altering the genotype, and it involves DNA methylation, chromatin remodeling, histone modification, micro RNAs, and broad spectrum of regulatory proteins [199]. Diet and different nutrient profiles were shown to regulate epigenetic pathways early in fetal and postnatal life, in a way that can predispose to several diseases including diabetes.

Cancer has been associated with inherited or acquired genetic mutations, but components of epigenetic signaling have also been recognized in its development, suggesting that epigenetic modifications may provide another plausible mechanism linking etiologies of cancer and diabetes [200, 201]. On the other hand, diabetes display a range of endocrinological deregulations likewise increased levels of bioavailable estrogens due to inhibited liver production of SHBP [202] or increased production of androgens in ovaries [203]. These factors may attribute to risk of breast and endometrial cancers [105, 106]. Expansion of adipose tissue, which is now perceived as highly active gland, is one of the key steps in development of DM. Adipocytes produce polypeptide hormones known as adipokines, which regulate glucose metabolism, insulin sensitivity, immune system, and angiogenesis. Leptin and adiponectin, two most intensively secreted adipokines, represent opposite metabolic activity. Leptin is positively associated with diabetes, obesity, and metabolic syndrome, but by its proangiogenic, antiapoptotic properties may exert a mitogenic influence on breast and pancreatic cancer cells [204]. In contrast, adiponectin acts as negative regulator of diabetes and was shown to inhibit cancer development potentially by pro-apoptotic, anti-inflammatory, and antiangiogenic activities [205–207]. It seems that adverse secretory profile of adipokines plays a direct role in increasing insulin resistance and may also be involved in favoring growth of cancer. Finally, it is of particular note to realize that carcinogenic factors related to DM may affect more individuals than it is expected. One-third of diabetic persons remain undiagnosed, and 35% of US adults have prediabetes with already developing metabolic derangements potentially promoting carcinogenesis. Epidemiologic reports suggest that the latter group is at higher risk of cancer.

Overall, the wide spectrum of DM pathophysiology involves insulin resistance, altered glucose, fat metabolism, chronic hormonal, inflammatory derangements, and persistent oxidative stress. Therefore diabetes is more likely to create environment leading to sustained cycles of cellular destruction and subsequent proliferation, where accumulation of random genetic errors contribute to metaplasia-dysplasia-carcinoma sequence.

## 8. Conclusions

Diabetes is a high-risk state for several diseases, with cancer now added to the list. In the context of globally exploding diabetes incidence, elucidating the diabetes and cancer association is an essential task. Given the complexity of reciprocal interaction of pathophysiological pathways underlying diabetes, risk of cancer may be increased by number of direct and indirect mechanisms. Further mechanistic studies are warranted to establish biological pathways linking both diseases and thereafter formulate efficient clinical preventive strategies and public health policies to avoid overlapping burden of both diseases that already have tremendous impact on public health and economy. 

## Figures and Tables

**Table 1:** Cancer risks in diabetes.

Study method (reference)	First author (year of publication)	Country	Sample	Followup duration	Risk of cancer among DM participants (95% CI or *P* value)
Prospective cohort [23]	Jee, 2005	Korea	1,298,385	10 years	Men HR = 1.24 (1.20–1.28) Women HR = 1.33 (1.25–1.41)
Prospective cohort [40]	Yeh, 2012	USA	18,280 (599 diabetic subjects)	17 years (exclusion of cancer cases in first 2 years)	HR = 1.22 (0.98–1.53)
Retrospective cohort [41]	Lee, 2012	Taiwan	985,815 (104,343 diabetic subjects)	11 years	RR = 1.56 (1.43–1.71)
Retrospective cohort [42]	Lo, 2012	Taiwan	895,434 in DM cohort and 895,434 in controls	13 years	HR = 1.19 (1.17–1.20)
Prospective cohort [43]	Hense, 2011	Germany	26,742 diabetic subjects	5 years	SIR = 1.14 (1.10 –1.21)
Retrospective cohort [44]	Geraldine, 2012	Belgium	17,746 (13,737 diabetic subjects)	Mean observation time: 5 years	HR=1.84 (1.51–2.24)
Retrospective cohort [45]	Zhang, 2012	China	7950 diabetic subjects	Mean observation time: 8 years	Men SIR = 1.331 (1.143–1.518) Women SIR = 1.737 (1.478–1.997)
Meta-analysis [38]	Noto, 2011	12 cohorts	257,222 diabetes subjects	—	Men RR = 1.14 (1.06–1.1.23) Women RR = 1.18 (1.08–1.28)
Meta-analysis [39]	Noto, 2010	4 cohort and 1 case-control study, all Japanese	250,479 subjects	—	OR = 1.70 (1.38–2.10)

**Table 2:** Summary of meta-analyses of specific cancer risk in diabetes.

Cancer type	Authors [reference]	Year of publication	No. of cohort/no. of case-control studies	Risk estimates (95% CI)
Liver	Wang et al. [47]	2012	25/0	SRR = 2.01 (1.61–2.51)
Pancreas	Ben et al. [64]	2011	35/0	RR = 1.94 (1.66–2.27)
Colon and rectum	Deng et al. [77]	2012	16/8	RR = 1.26 (1.20–1.31)
Esophagus	Huang et al. [86]	2012	11/6	SSR = 1.30 (1.12–1.50)
Kidney	Larsson and Wolk [88]	2011	9/0	RR = 1.42 (1.06–1.91)
Bladder	Larsson et al. [96]	2006	9/7	RR = 1.24 (1.08–1.42)
Breast	Liao et al. [103]	2011	7/5	RR = 1.25 (1.20–1.29)
Endometrium	Friberg et al. [104]	2007	3/13	RR = 2.1 (1.75–2.53)
Blood	Castillo et al. [107]	2012	13/13	NHL OR = 1.22 (1.07–1.39) Leukemia OR = 1.22 (1.03–1.44) Myeloma OR = 1.22 (0.98–1.53)
